# The patient’s perspective on precautionary labelling

**DOI:** 10.1186/2045-7022-1-S1-S42

**Published:** 2011-08-12

**Authors:** Audrey DunnGalvin

**Affiliations:** 1 University College Cork, Cork, Ireland

## Background

We have previously shown that uncertainty is a core theme in living and coping with food allergy. Uncertainty about labelling may result in anxiety/avoidant behaviour or frustration/risky behaviours. We aimed to evaluate understanding and acceptance of defined thresholds and risk stratification in parents and teens.

## Method

Under the aegis of FARRP, we carried out focus groups with parents of children and teens with food allergy (N30), teens with food allergy (N20), and clinicians. Data was analysed using grounded theory.

## Results

The analysis revealed 4 main themes : 'The reality of living with risk'; 'maintaining a balance'; 'feeling Informed and in control of risk management'; 'communicating thresholds'. Current risk hazard approaches reinforce uncertainty and that consumers and clinicians want a 'new' way of risk assessment and management.

## Conclusion

The themes will form a basis for further discussion with parents, children, teens and adults living with food allergy, and the health professionals who diagnose and advise them. Consumer engagement can ensure communication tools and methods are valid and relevant to both majority groups and sub-groups.

